# Mechanistic and Clinical Overview Cardiovascular Toxicity of BRAF and MEK Inhibitors: *JACC: CardioOncology* State-of-the-Art Review

**DOI:** 10.1016/j.jaccao.2022.01.096

**Published:** 2022-03-15

**Authors:** Claire Glen, Yun Yi Tan, Ashita Waterston, Thomas R. Jeffry Evans, Robert J. Jones, Mark C. Petrie, Ninian N. Lang

**Affiliations:** a Institute of Cardiovascular and Medical Sciences, University of Glasgow, Glasgow, United Kingdom; b Beatson West of Scotland Cancer Centre, NHS Greater Glasgow and Clyde, Glasgow, United Kingdom; c Institute of Cancer Sciences, University of Glasgow, Glasgow, United Kingdom

**Keywords:** BRAF inhibitor, cardio-oncology, cardiovascular toxicity, hypertension, left ventricular systolic dysfunction, MEK inhibitor, melanoma, ACE, angiotensin-converting enzyme, AF, atrial fibrillation, BRAF, rapidly accelerated fibrosarcoma B-type, CVAE, cardiovascular adverse event, EGFR, epidermal growth factor receptor, ERK, extracellular signal-regulated kinase, LVSD, left ventricular systolic dysfunction, MEK, mitogen-activated extracellular signal-regulated kinase, RAF, rapidly accelerated fibrosarcoma, VEGF, vascular endothelial growth factor

## Abstract

Rapidly accelerated fibrosarcoma B-type (BRAF) and mitogen-activated extracellular signal-regulated kinase (MEK) inhibitors have revolutionized melanoma treatment. Approximately half of patients with melanoma harbor a BRAF gene mutation with subsequent dysregulation of the RAF-MEK-ERK signaling pathway. Targeting this pathway with BRAF and MEK blockade results in control of cell proliferation and, in most cases, disease control. These pathways also have cardioprotective effects and are necessary for normal vascular and cardiac physiology. BRAF and MEK inhibitors are associated with adverse cardiovascular effects including hypertension, left ventricular dysfunction, venous thromboembolism, atrial arrhythmia, and electrocardiographic QT interval prolongation. These effects may be underestimated in clinical trials. Baseline cardiovascular assessment and follow-up, including serial imaging and blood pressure assessment, are essential to balance optimal anti-cancer therapy while minimizing cardiovascular side effects. In this review, an overview of BRAF/MEK inhibitor–induced cardiovascular toxicity, the mechanisms underlying these, and strategies for surveillance, prevention, and treatment of these effects are provided.

## Highlights

• BRAF and MEK inhibitors are associated with adverse cardiovascular events.
• Hypertension and LVSD are most common, and incidence is higher with combination therapy.
• Understanding the incidence and mechanisms of BRAF/MEK inhibitor–associated cardiovascular toxicity will aid the refinement of strategies for cardiotoxicity prevention and treatment.

Cancer outcomes have improved considerably over the past 2 decades. Approximately 50% of patients with a diagnosis of cancer in any form will now survive at least 10 years.^1^ These improvements have, to a large extent, been caused by the rapid introduction of novel cancer therapies. As survivorship has improved, concerns about cardiovascular toxicity have become increasingly relevant. In addition to defining the manifestations and incidence of cardiovascular toxicity, understanding the mechanisms of the adverse cardiovascular events has become a focus of research. This may allow the identification of patients at higher risk of adverse cardiovascular effects and should lead to the development of better strategies for their prevention and treatment.

The prognosis for patients with metastatic cutaneous melanoma has, historically, been very poor. Immunotherapy and targeted therapies have transformed the outlook for many of these patients.^2^ These options are now available for the palliative treatment of patients with unresectable stage III or stage IV (metastatic) disease and as adjuvant therapy, with curative intent, after complete resection of stage III disease.^2^^,^^3^ Activating mutations of the rapidly accelerated fibrosarcoma B-type (BRAF) gene are found in approximately 60% of cutaneous melanomas,^4^ and the therapeutic potential of BRAF inhibition has been harnessed. Vemurafenib, the first BRAF inhibitor approved for the treatment of melanoma, was associated with a 63% reduction in the risk of death compared with dacarbazine chemotherapy.^5^ However, inhibition of BRAF alone was associated with the emergence of drug-resistance as a result of paradoxical hyperactivation of mitogen-activated extracellular signal-regulated kinase (MEK), the signaling molecule immediately downstream of BRAF in this pathway. Therefore, concomitant inhibition of BRAF and MEK has been found to bring about more prolonged disease control.^6^

In conjunction with their potent anti-cancer effects, cardiovascular toxicity associated with BRAF and MEK inhibition is increasingly recognized. These adverse cardiovascular effects include systemic hypertension, left ventricular systolic dysfunction (LVSD) and heart failure as well as venous thromboembolism, atrial fibrillation (AF), and QT interval prolongation. These effects are likely to be the consequence of the overlap between pathogenetic cancer target pathways and homeostatic pathways required for normal cardiac and vascular physiology.^7^, ^8^, ^9^, ^10^ We review the current evidence and understanding of the incidence and mechanisms of BRAF inhibitor/MEK inhibitor–associated cardiovascular toxicity. We provide an overview of surveillance and management strategies for patients with, or at risk of, BRAF inhibitor/MEK inhibitor–associated cardiovascular toxicity.

## BRAF and MEK Inhibition In Cancer Therapy: A Primer

The most common BRAF mutation, V600E, reflects the substitution of valine to glutamine at codon 600 in exon 15, which results in constitutive activation of BRAF’s kinase function.^11^ Since 2005, 3 BRAF inhibitors have been approved for use in patients with BRAF mutant melanoma—first, vemurafenib and, subsequently, dabrafenib and encorafenib. The use of these drugs as monotherapy brought a significant increase in progression-free and overall survival for patients with advanced melanoma. However, approximately 50% of patients developed acquired resistance, limiting the benefits of this intervention, even in patients whose disease was initially sensitive to the drugs.^12^ Subsequent efforts focused on the development of novel MEK inhibitors that now include trametinib, cobimetinib, and binimetinib. Three combinations of BRAF inhibitor/MEK inhibitor are approved for use (Table 1) in the treatment of advanced BRAF mutant melanoma. Combination therapy with BRAF inhibitor and MEK inhibitor results in a reduced incidence of BRAF inhibitor–induced skin tumors,^13^ delays the emergence of resistance, and is associated with prolonged progression-free and overall survival when compared with BRAF inhibitor monotherapy (Table 2).^14^, ^15^, ^16^

**Table 1:** Summary of BRAF and MEK Inhibitors Commonly Used to Treat Melanoma

BRAF Inhibitor	MEK Inhibitor	Approved Combinations
Dabrafenib	Trametinib	Dabrafenib + Trametinib
Vemurafenib	Cobimetinib	Vemurafenib + Cobimetinib
Encorafenib	Binimetinib	Encorafenib + Binimetinib

BRAF = rapidly accelerated fibrosarcoma B-type; MEK = mitogen-activated extracellular signal-regulated kinase.

**Table 2:** Outcomes of Patients With Unresectable Stage III/IV Melanoma Treated With BRAF Inhibitor/MEK Inhibitor Versus BRAF Inhibitor Monotherapy

	COMBI-d Trial^16^^,^^24^		coBRIM Trial^55^		COLUMBUS Trial^56^	
	Dabrafenib	Dabrafenib + Trametinib	Vemurafenib	Vemurafenib + Cobimetinib	Encorafenib	Encorafenib + Binimetinib
Objective response rate**,** %	55	67	50	70	51	63
Complete response rate**,** %	15	18	10	16	9	16
Median PFS, mo	8.8	11.0	7.2	12.3	9.6	14.9
Median OS, mo	18.7	25.1	17.4	22.3	23.5	33.6

coBRIM = Cobimetinib combined with vemurafenib in advanced BRAFV600-mutant melanoma; COLUMBUS = Encorafenib plus binimetinib versus vemurafenib or encorafenib in patients with BRAF-mutant melanoma; COMBI-d = Combined BRAF and MEK inhibition versus BRAF inhibition alone in melanoma; OS = overall survival; PFS = progression-free survival; other abbreviations as in Table 1.

While immunotherapy with T cell checkpoint inhibitors, targeting PD(L)-1 alone or combined with anti-CTLA-4 antibodies, remains first line treatment for patients with BRAF wild-type advanced (unresectable stage III or stage IV) cutaneous melanoma,^17^^,^^18^ treatment options for patients with BRAF-mutated melanoma include immunotherapy or combined BRAF inhibitor and MEK inhibitor. There have been no clinical trials directly comparing the effectiveness of immunotherapy versus BRAF inhibitor and MEK inhibitor in BRAF-mutant advanced melanoma; therefore, the best sequence of therapy in this patient group remains unknown. In practice, targeted therapy is generally offered as first line for patients with a high symptom and tumor burden, as treatment responses tend to be more rapid than those achieved using immunotherapy.^17^ Patients may ultimately receive both treatments.^17^ More recently, the combination of the anti-PD-L1monoclonal antibody, atezolizumab, with combined BRAF and MEK inhibition (vemurafenib + cobimetinib) significantly prolonged progression-free survival compared with the combination of vemurafenib and cobimetinib, plus placebo in patients with advanced unresectable BRAF mutation–positive cutaneous melanoma.^19^

Following the earliest demonstration of the clinical effectiveness of BRAF- and MEK-targeted therapies in patients with advanced unresectable melanoma,^14^^,^^20^ their use has broadened to include their use in the adjuvant setting for treatment, with curative intent, of patients with resected stage III disease.^2^^,^^3^ Indeed, in this group adjuvant treatment with dabrafenib plus trametinib for 12 months is associated with a 53% lower risk of relapse and an estimated overall survival at 3 years of 86% compared with 77% in patients receiving placebo.^21^

While the current primary role for BRAF inhibitor and MEK inhibitor is in the treatment of melanoma,^15^^,^^22^, ^23^, ^24^ these agents have also been used in the treatment of other cancers including non-small cell lung cancer^25^ and colorectal cancer.^26^ Although combination treatment with dabrafenib and trametinib is an approved therapy for BRAF mutant non-small cell lung cancer, the prevalence of BRAF mutations in this group is only 3% to 5%.^27^ BRAF mutations are present in approximately 10% of colorectal cancers, again raising the possibility of using BRAF inhibitor and MEK inhibitor. Initial clinical trials of BRAF inhibitors in patients with colorectal cancers were disappointing with a phase II study of vemurafenib reporting a response rate of only 5%.^28^ Preclinical models of BRAF^V600E^ mutated colorectal cancer have shown that BRAF inhibition causes rapid feedback activation through the epidermal growth factor receptor (EGFR). Combining BRAF and MEK inhibitors (encorafenib and binimetinib) with the anti-EGFR antibody, cetuximab, results in higher responses and a significantly longer overall survival compared with standard therapy in patients with metastatic colorectal cancers with the V600E BRAF mutation.^29^

### Key Points

• Approximately 60% of cutaneous melanomas harbor an activating BRAF gene mutation.
• Combination BRAF and MEK inhibitors are used for the treatment of unresectable metastatic melanoma and as adjuvant therapy.
• BRAF and MEK inhibitors are associated with adverse cardiovascular effects.

## Preclinical Insights to Cardiovascular Ras-RAF-MEK-ERK Signaling

The MAPK pathway is a key regulator of normal cell growth and proliferation. This signaling cascade is initiated by the binding of an extracellular ligand to specific membrane-bound receptor tyrosine kinases. Following the stimulation and phosphorylation of GFR adaptor protein, Src homology 2 domain containing protein becomes associated with the activated GFR with subsequent recruitment of downstream signaling proteins culminating in activation of Ras, the membrane-bound guanosine diphosphate to guanosine triphosphate (GTP) exchange protein (Central Illustration).^30^^,^^31^

**Central Illustration:**
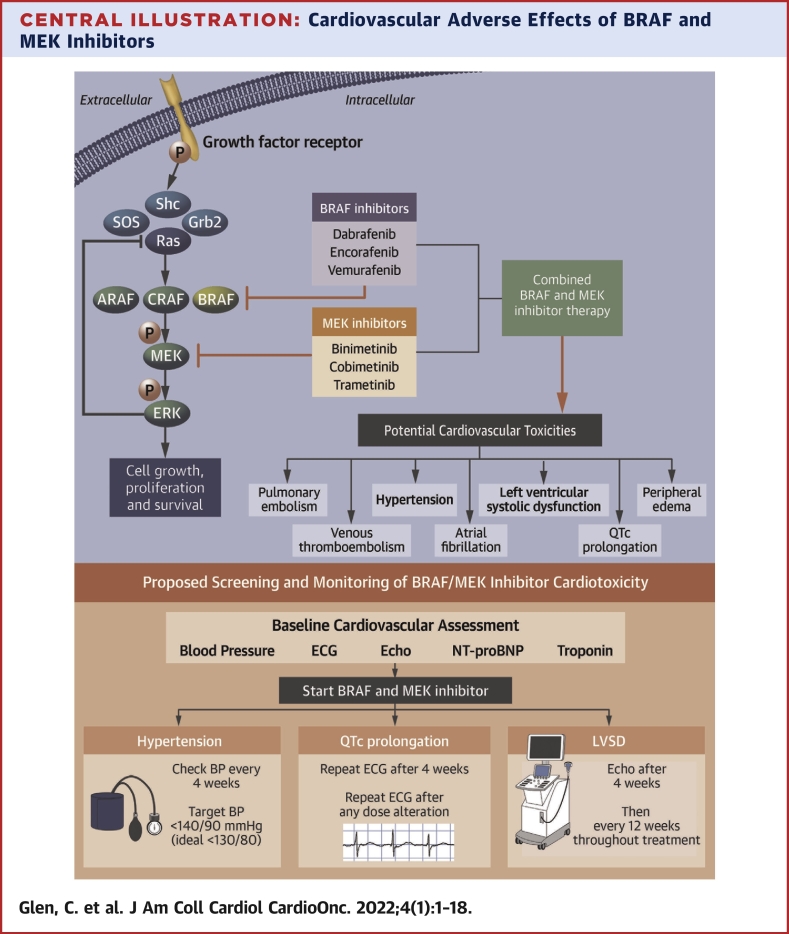
Cardiovascular Adverse Effects of BRAF and MEK Inhibitors The Ras-RAF-MEK-ERK signaling pathway plays an essential role in the regulation of numerous cellular activities. Targeting this pathway with rapidly accelerated fibrosarcoma B-type (BRAF) and mitogen-activated extracellular signal-regulated kinase (MEK) blockade results in control of cell proliferation and disease activity in certain BRAF-mutant cancers. BRAF and MEK inhibitors are associated with adverse cardiovascular effects including left ventricular systolic dysfunction (LVSD), hypertension, venous thromboembolism, and atrial arrhythmia. We propose strategies for the surveillance and treatment of these adverse effects. BP = blood pressure; ECG = electrocardiography; Echo = echocardiography; LVSD = left ventricular systolic dysfunction; NT-proBNP = N-terminal pro–B-type natriuretic peptide.

GTP-bound Ras interacts with rapidly accelerated fibrosarcoma (RAF) kinases leading to a cascade of phosphorylation events. Of the 3 RAF serine/threonine kinases (cRAF-1, ARAF, and BRAF), BRAF is the most potent inducer of phosphorylation and activation of MEK1 and MEK2 dual-specificity protein kinases.^32^ Activated MEK1/2 leads to the phosphorylation and activation of extracellular signal-regulated kinases (ERKs) 1 and 2.^33^^,^^34^ Activated ERK proteins provoke the phosphorylation and activity of numerous important substrates including GFRs, cytokines, transcription factors, and cell cycle regulator proteins. Accordingly, the RAF-MEK-ERK pathway is critically involved in the regulation of key cellular activities including proliferation, migration, angiogenesis, and suppression of apoptosis. Consequently, mutations of genes coding for proteins in this pathway may allow these processes to become dysregulated and constitutively activated—a feature common to many cancers.^35^

### Cardiac Ras-RAF-MEK-ERK signaling

ERK1 and ERK2 play important roles in cardiac development at a number of stages, reflecting their role in growth factor signaling and in the regulation of the cellular processes described previously.^36^ Genetic deletion of ERK1 in mice does not affect cardiac development, while deletion of ERK2 is fatal, caused by a defect in trophoblast development.^37^ Interactions between the ERK pathway and other signaling pathways such as protein kinase B and bone morphogenic protein may play important roles in cardiac development.^36^

The Ras-RAF-MEK-ERK pathway is a key component in processes including myocyte hypertrophy, cardiac remodeling and myocardial cell death.^36^ Cardiac hypertrophy occurs in response to a number of different stimuli including mechanical overload, oxidative stress, and neurohormonal stimulation. While this may be a physiological response to enhance contractility and cardiac output in response to increased demand (eg, “athlete’s heart”), it may also be pathological and result in the development of heart failure (eg, hypertensive heart disease). There is compelling evidence for the importance of the Ras-RAF-MEK-ERK signaling pathway in the development of cardiac hypertrophy. In animal models, transgenic expression of constitutively active Ras in mice results in cardiomyocyte hypertrophy but not in increased cardiac fibrosis.^38^ Both BRAF and cRAF-1 kinase activity have also been demonstrated to be essential for cardiomyocyte hypertrophy both in vitro and in vivo.^39^, ^40^, ^41^ MEK1-ERK1/2 signaling induces hypertrophy in both cultured myocytes and in hearts from transgenic mice but the downstream mechanisms stimulating this growth are poorly understood.^42^ Endomyocardial biopsies from patients with hypertrophic cardiomyopathy reveal that the expression of H-RAS positively correlates with the severity of hypertrophy.^43^ Patients with Noonan and Leopard syndromes, as well as other autosomal dominant disorders with increased Ras-RAF-MEK-ERK activity, exhibit a hypertrophic cardiomyopathy phenotype.^44^ In adults with a LV assist device, the subsequent beneficial reverse cardiac remodeling and reduction in myocyte hypertrophy is associated with decreased ERK activity.^36^

Members of the MAPK family have cardioprotective effects, largely considered to be caused by antagonistic effects on apoptotic regulatory pathways. In vivo studies assessing loss of cardiac cRAF-1 activity revealed greater apoptosis in the resting state as well as in response to pressure overload in comparison with wild-type animals.^39^ The MEK1-ERK2 signaling pathway is required for the protection of the myocardium following ischemic injury in vivo and heterozygote ERK2 gene–targeted mice have greater myocardial injury following ischemia-reperfusion injury induced via ligation of left anterior descending artery than wild-type mice.^45^ Although the role of ERK pathway activation in cardioprotection has been studied extensively, the precise mechanism by which it evokes this effect is less well understood and is likely to involve multiple processes. This may include interleukin-10 activation of ERK1/2 to inhibit tumor necrosis factor-α–induced apoptosis,^46^ and the suppression of gap junction permeability as a result of mitochondrial potassium-sensitive adenosine triphosphate channel opening in ischemic myocardium.^47^

### Vascular Ras-RAF-MEK-ERK signaling

The Ras-RAF-MEK-ERK pathway plays a protective role in the vasculature through interaction with vascular endothelial growth factor (VEGF). In cultured endothelial cells, phosphorylation of VEGF tyrosine kinase receptor 2 activates phospholipase C with consequent activation of the RAF-MEK-ERK pathway. This results in increased endothelial cell proliferation.^48^ Activation of ERK1/2 by phospholipase C also activates endothelial nitric oxide (NO) synthase and stimulates release of prostacyclin mediated by protein kinase C.^49^^,^^50^ This results in vasodilation in endothelial cells and has antiproliferative effects in smooth muscle cells. The RAF-MEK-ERK pathway is also involved in angiogenesis, the formation of new blood vessels, via interaction with fibroblast growth factor and platelet-derived growth factors.^51^

### Key Points

• The Ras-RAF-MEK-ERK pathway is a key regulator of normal cell growth and proliferation.
• The Ras-RAF-MEK-ERK pathway plays a key role in myocyte hypertrophy, cardiac remodeling, and myocardial cell death.
• The Ras-RAF-MEK-ERK pathway interacts with VEGF, fibroblast growth factor, and platelet-derived growth factors to stimulate endothelial cell proliferation and angiogenesis in the vasculature.

## Cardiovascular Toxicity of BRAF and MEK Inhibitors in Patients with Cancer

**Figure 1:**
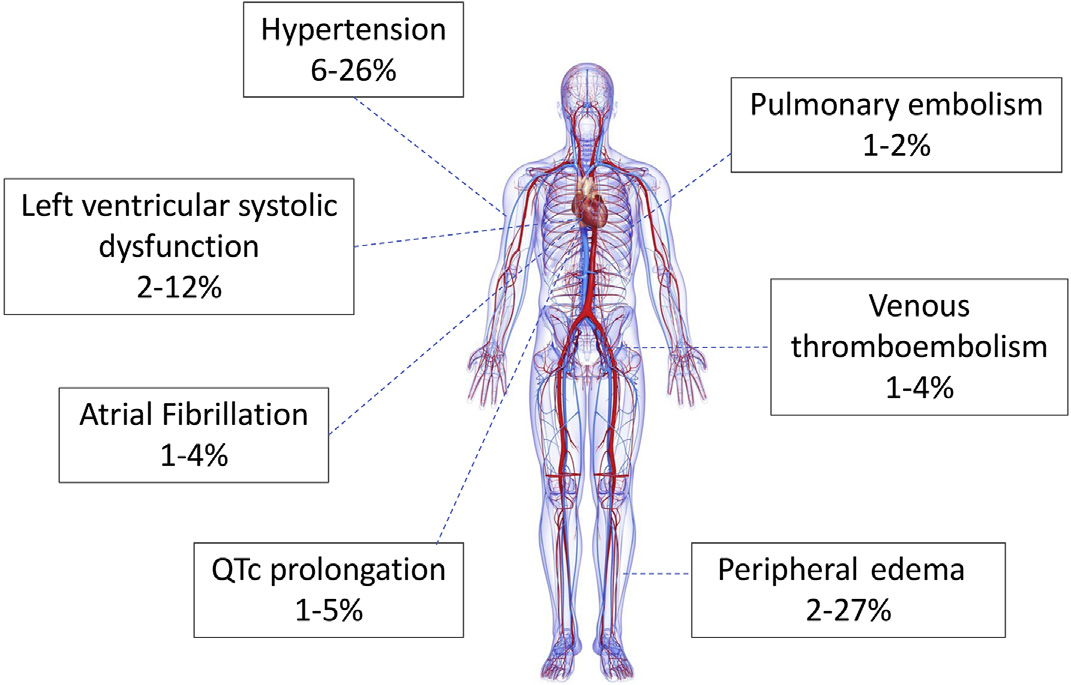
Incidence of BRAF Inhibitor/MEK Inhibitor–Associated Cardiovascular Adverse Events From Clinical Trials Rapidly accelerated fibrosarcoma B-type **(**BRAF) and mitogen-activated extracellular signal-regulated kinase (MEK) inhibitor treatment is associated with adverse cardiovascular effects. Included are the most commonly reported cardiovascular adverse events and their estimated incidence from clinical trials.^9^^,^^16^^,^^55^^,^^56^^,^^79^, ^80^, ^81^

Given the central role of the Ras-RAF-MEK-ERK pathway in cardiac and vascular physiology, its pharmacological manipulation might be expected to have unwanted cardiovascular effects. Indeed, BRAF inhibitor/MEK inhibitor treatment is associated with LVSD, systemic hypertension, atrial arrhythmia, QT interval prolongation, and venous thromboembolism (Figure 1).

### LVSD and heart failure

#### Incidence of BRAF inhibitor/MEK inhibitor–associated LV dysfunction and heart failure

Cardiovascular adverse events (CVAEs) associated with BRAF and MEK inhibitors have been poorly captured in clinical trials. The incidence of LVSD in patients treated with BRAF inhibitor and MEK inhibitor in clinical trials, with a median follow-up of 9 to 16 months, has been reported as 2% to 12%.^9^^,^^10^ However, this may be an underestimate for several reasons. There is inconsistent reporting of heart failure in clinical trials^52^ and lack of a standardized definition of LVSD related to anti-cancer therapies. Reduction in LV ejection fraction (LVEF) in trials has been defined using the Common Terminology Criteria for Adverse Events (CTCAE) definitions.^53^ To meet CTCAE definitions of ejection fraction decreased, the LVEF must be below 50% and with at least a 10% decline from baseline assessment. However, the CTCAE also includes separate (and frequently overlapping) criteria for symptomatic heart failure as well as another category for LVSD. Oncology trial investigators may choose to classify a given event under ejection fraction decreased, LVSD, or heart failure with associated grades. This contributes to difficulty in comparing results from different trials and the effects of different cancer therapies. Furthermore, patients with significant underlying cardiovascular morbidity are commonly excluded from clinical trials.^54^ Indeed, all of the trials outlined in Table 2 excluded patients with clinically significant cardiac dysfunction or cardiovascular disease, or a history or evidence of cardiovascular risk including uncontrolled hypertension, prolonged QTc interval, heart failure (New York Heart Association functional class II and above), and those with an acute coronary syndrome or angioplasty within the preceding 6 months. While regular electrocardiograms were recorded, routine serial echocardiography was not performed in the early trials. Adverse events of shortness of breath and peripheral edema were reported; however, in the absence of echocardiography, it is unknown whether these symptoms indicated LVSD and heart failure. More recent trials including Flaherty et al,^14^ the COMBI-d study (Combined BRAF and MEK inhibition versus BRAF inhibition alone in melanoma),^16^ the COMBI-v study (Dabrafenib Plus Trametinib vs Vemurafenib Alone in Unresectable or Metastatic BRAF V600E/K Cutaneous Melanoma),^22^ coBRIM trial (Cobimetinib combined with vemurafenib in advanced BRAFV600-mutant melanoma),^55^ and COLUMBUS study (Encorafenib plus binimetinib versus vemurafenib or encorafenib in patients with BRAF-mutant melanoma)^56^ all included echocardiography at baseline, after 4 weeks, and at 12-weekly intervals thereafter. The COLUMBUS study included measurement of creatinine kinase and troponin at each treatment cycle, but to date, no studies have measured N-terminal pro–B-type natriuretic peptide, a cardiac biomarker now used to diagnose heart failure.^57^ For all of these reasons, the reported incidence of LVSD or heart failure is likely to be an underestimate of the “real-world” population. Long-term follow-up is absent, and patients with asymptomatic cardiovascular toxicity may be underrecognized. This is an increasing concern as patients with cancer now survive longer and receive treatment over longer periods, especially as these medications are increasingly used with curative intent in the adjuvant setting, in which many patients will have near normal life expectancy.

Table 3 summarizes CVAEs reported in phase 2 and phase 3 clinical trials of BRAF inhibitors and MEK inhibitors. Several studies have reported cardiotoxic effects associated with BRAF inhibitor and MEK inhibitor monotherapy. In a meta-analysis of 12 randomized placebo controlled trials (9 MEK inhibitor monotherapy studies, 1 BRAF inhibitor monotherapy study, and 2 combination therapy studies), MEK inhibitors were associated with a higher risk of reduced LVEF (OR: 3.35; 95% CI: 1.58-7.07) and peripheral edema (OR: 2.87; 95% CI: 1.93-4.27) when compared with placebo.^58^ It should be noted that clinical trial reports of peripheral edema are otherwise undefined. While there may be alternative explanations for peripheral edema in patients with cancer, it is likely that at least some of these patients have undiagnosed heart failure. Combination therapy with BRAF inhibitor/MEK inhibitor was associated with a higher risk of ejection fraction decrease compared with BRAF inhibitor plus placebo (OR: 2.48; 95% CI: 1.46-4.21).^58^ Therefore, reduction in LVEF is more common in patients treated with combination BRAF inhibitor/MEK inhibitor therapy. In the COMBI-d study, reduced LVEF was reported in 4% and peripheral edema in 14% of patients treated with dabrafenib and trametinib compared with 2% and 5%, respectively, in the dabrafenib-only group.^16^ Similarly, in the coBRIM trial, reduced LVEF was reported in 12% of patients in the vemurafenib plus cobimetinib arm compared with 5% in the vemurafenib-only arm.^55^ In patients treated with encorafenib and binimetinib in the COLUMBUS trial, LV dysfunction was reported in 8% of patients compared with 2% in the encorafenib-only group.^56^

**Table 3:** Incidence of Reported Cardiovascular Adverse Events Caused by BRAF and MEK Inhibitors in Clinical Trials

First Author/Study	Cancer Type	Treatment Arm	Grade of AE	Hypertension	Decreased Ejection Fraction	Peripheral Edema	QTc Prolongation	AF	VTE
Flaherty et al, 2012^14^	Metastatic melanoma with BRAF V600 mutation	Dabrafenib and trametinib (n = 54)	All grade	6 (7)	6 (7)	27 (29)	Not reported	Not reported	Not reported
			≥3	1 (1)	1 (1)	0	Not reported	Not reported	Not reported
		Dabrafenib and placebo (n = 54)	All grade	4 (2)	0	17 (9)	Not reported	Not reported	Not reported
			≥3	0	0	0	Not reported	Not reported	Not reported
COMBI d; Long et al, 2014^16^	Unresectable stage IIIc or stage IV melanoma with BRAF V600 mutation	Dabrafenib and trametinib (n = 211)	All grade	22 (46)	4 (9)	14 (30)	Not reported	Not reported	Not reported
			≥3	4 (8)	<1 (1)	<1 (1)	Not reported	Not reported	Not reported
		Dabrafenib and placebo (n = 212)	All grade	14 (29)	2 (5)	5 (10)	Not reported	Not reported	Not reported
			≥3	5 (10)	<1 (1)	<1 (1)	Not reported	Not reported	Not reported
COMBI-v; Robert et al, 2015^22^	Metastatic melanoma with BRAF V600 mutation	Dabrafenib and trametinib (n = 352)	All grade	26 (92)	8 (29)	12 (42)	Not reported	Not reported	Not reported
			≥3	14 (48)	4 (13)	<1 (1)	Not reported	Not reported	Not reported
		Vemurafenib (n = 352)	All grade	24 (84)	0	10 (35)	Not reported	Not reported	Not reported
			≥3	9 (32)	0	<1 (1)	Not reported	Not reported	Not reported
COMBI-AD; Long et al, 2017^21^	Stage III melanoma with complete resection and BRAF V600 mutation	Dabrafenib and trametinib (n = 438)	All grade	11 (49)	Not reported	13 (58)	Not reported	Not reported	Not reported
			≥3	6 (25)	Not reported	<1 (1)	Not reported	Not reported	Not reported
		Placebo (n = 432)	All grade	8 (35)	Not reported	4 (19)	Not reported	Not reported	Not reported
			≥3	2 (8)	Not reported	0	Not reported	Not reported	Not reported
coBRIM; Larkin et al, 2014^15^	Unresectable stage IIIc or stage IV melanoma with BRAF V600 mutation	Vemurafenib and cobimetinib (n = 247)	All grade	Not reported	8 (19)	Not reported	4 (9)	Not reported	Not reported
			≥3	Not reported	1 (3)	Not reported	<1 (1)	Not reported	Not reported
		Vemurafenib and placebo (n = 248)	All grade	Not reported	3 (7)	Not reported	5 (13)	Not reported	Not reported
			≥3	Not reported	1 (3)	Not reported	1 (3)	Not reported	Not reported
coBRIM (update); Ascierto et al, 2016^55^	Unresectable stage IIIc or stage IV melanoma with BRAF V600 mutation	Vemurafenib and cobimetinib (n = 247)	All grade	16 (39)	12 (29)	14 (34)	5 (11)	4 (9)	1 (3)
			≥3	6 (15)	2 (5)	0	1 (3)	1 (3)	<1 (2)
		Vemurafenib and placebo (n = 248)	All grade	8 (20)	5 (13)	11 (28)	5 (13)	1 (3)	<1 (2)
			≥3	3 (7)	1 (3)	<1 (1)	1 (3)	0	<1 (1)
COLUMBUS; Dummer et al, 2018^56^	Unresectable stage IIIb, IIIc or stage IV melanoma with BRAF V600 mutation	Encorafenib and binimetinib (n = 192)	All grade	11 (21)	6 (11)	2 (3)	0	<1 (1)	4 (7)
			≥3	6 (11)	1 (2)	0	0	<1 (1)	1 (2)
		Encorafenib (n = 194)	All grade	6 (11)	2 (4)	2 (3)	4 (7)	1.5 (3)	2 (4)
			≥3	3 (6)	1 (2)	0	<1 (1)	0	<1 (1)
		Vemurafenib (n = 191)	All grade	11 (21)	<1 (1)	4 (7)	3 (6)	1.5 (3)	1 (2)
			≥3	3 (6)	0	<1 (1)	0	0	1 (2)

Values are % (n).

AE = adverse event; AF = atrial fibrillation; COMBI-v = Dabrafenib Plus Trametinib vs Vemurafenib Alone in Unresectable or Metastatic BRAF V600E/K Cutaneous Melanoma; VTE = venous thromboembolism; other abbreviations as in Tables 1 and 2.

A higher risk of cardiotoxicity with combination BRAF inhibitor/MEK inhibitor therapy is also suggested in a subsequent meta-analysis^9^ including 5 randomized clinical trials with a total of 2,317 patients with melanoma treated with BRAF inhibitor and MEK inhibitor. The combination of BRAF inhibitor and MEK inhibitor was associated with a >3-fold increased risk of a reduction in LVEF (relative risk [RR]: 3.72; 95% CI: 1.74-7.95) compared with BRAF inhibitor monotherapy. A total of 8.1% of patients in the combination treatment group had a reduction in LVEF compared with 2% in the BRAF inhibitor monotherapy group. A recent retrospective, observational, nonrandomized study sought to examine cardiovascular events in a real-world setting.^59^ This cross-sectional analysis of the Food and Drug Administration’s adverse events reporting system and longitudinal analysis of Truven Health Analytics/IBM MarketScan database collected data on reporting of CVAEs for patients on BRAF inhibitor monotherapy and BRAF inhibitor/MEK inhibitor combination therapy. A total of 657 patients with cancer were included (of whom 88.7% had melanoma) with a median follow-up time of 207 (IQR: 85-476) days. A total of 185 (28.2%) patients experienced a CVAE during the follow-up period. Heart failure, as defined by the International Classification of Diseases–10th Edition coding, was reported in 2.7% of patients. Cross-sectional pharmacovigilance analysis included 7,712 adverse events, of which 745 were cardiovascular. Combination therapy was associated with a higher risk of heart failure (OR: 1.62; 95% CI: 1.14-2.30) in comparison with BRAF inhibitor monotherapy.^59^ This analysis specifically only included patients with symptomatic heart failure, in comparison with prior meta-analyses that are less clear about the distinction between asymptomatic declines in LVEF and symptomatic heart failure. This further highlights the problems associated with a lack of universal definition of heart failure in cancer trials.

Patient factors that may predispose to BRAF/MEK inhibitor–associated cardiotoxicity include pre-existing cardiovascular disease (prior heart failure/cardiomyopathy, ischemic heart disease, and severe valvular heart disease) as well as advancing age and other conventional cardiovascular risk factors such as hypertension, diabetes mellitus, chronic kidney disease, and cigarette smoking. These are included in a cardiovascular risk stratification proforma for patients treated with combined BRAF/MEK inhibitor published in a recent International Cardio-Oncology Society endorsed position statement from the European Society of Cardiology.^60^ Prior exposure to other potentially cardiotoxic chemotherapy or radiotherapy should also be considered as risk factors.^60^ These components would be considered to be risk factors for most cancer therapy–related cardiotoxicity^61^ and research to identify more specific risk factors for BRAF inhibitor/MEK inhibitor–induced effects is overdue.

#### Assessment and management of LV function before and during BRAF inhibitor/MEK inhibitor treatment

Prior to treatment with BRAF inhibitor/MEK inhibitor, echocardiography is recommended to assess baseline cardiac function.^62^ Following the commencement of BRAF inhibitor/MEK inhibitor therapy echocardiography is recommended after 4 weeks and at 3-month intervals thereafter throughout treatment. This recommendation is based upon the Summary of Product Characteristics package inserts for these drugs.^63^, ^64^, ^65^, ^66^, ^67^, ^68^ The European Society of Cardiology position paper on cancer treatments and cardiovascular toxicity^69^ does not give specific recommendations related to BRAF inhibitor/MEK inhibitor. However, this frequency of echocardiography surveillance is in line with the recommendations for monitoring for cardiotoxic effects of anti-HER2 therapies, such as trastuzumab.

An asymptomatic decline in LVEF of ≥10% to an absolute value of <50% is considered to be evidence of cardiotoxicity. A decline in LVEF by <10% from baseline and to 40% to 49% may also reflect cardiotoxicity but the diagnostic certainty of this is less clear, unless accompanied by an associated worsening in LV global longitudinal strain by >15% from baseline. We recognize that this echocardiographic measurement is not routinely performed in all centers but strongly encourage its use. Any new reduction in LVEF to <40% or the development of symptoms or signs of heart failure is considered to be “severe” cardiotoxicity. These definitions are in keeping with a recent consensus statement from the International Cardio-Oncology Society.^70^ The safety of BRAF inhibitor and MEK inhibitor use in patients with a baseline LVEF that is either below 50% or the institutional lower limit of normal has not been established and therefore these drugs should be used with caution for this patient group.^63^, ^64^, ^65^, ^66^, ^67^, ^68^

**Figure 2:**
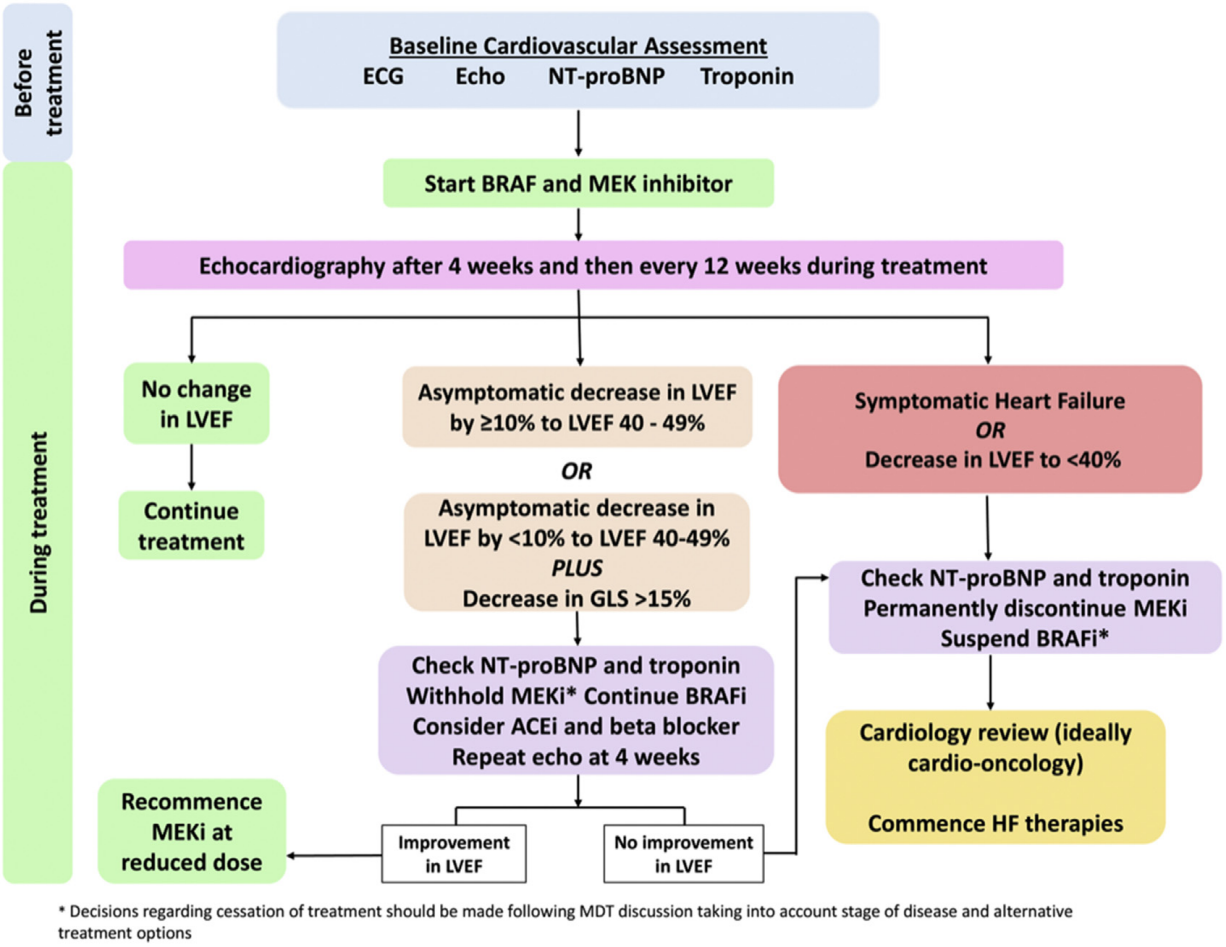
Proposed Pathway for the Management of BRAF Inhibitor/MEK Inhibitor–Associated LVSD There are no international guidelines for the management of BRAF and MEK inhibitor–associated left ventricular systolic dysfunction (LVSD). We propose a pathway for the treatment of LVSD based on echocardiographic surveillance of LV ejection fraction (LVEF). These suggestions are evolved from previous management algorithms and are consistent with our own practice. Decisions should be made following discussion between oncology and cardiology and taking into account stage of disease and alternative treatment options. ACEi = angiotensin-converting enzyme inhibitor; BRAFi = BRAF inhibitor; ECG = electrocardiography; Echo = echocardiography; HF = heart failure; LLN = lower limit of normal; MDT = multidisciplinary team; MEKi = MEK inhibitor; NT-proBNP = N-terminal pro**–**B-type natriuretic peptide; other abbreviations as in Figure 1.

We propose a pathway for the management of BRAF inhibitor– and MEK inhibitor–associated LVSD (Figure 2). Importantly, these suggestions are made in the absence of international guidelines, evolved from previous management algorithms,^10^ and are consistent with our own practice. All decisions should be made jointly by oncology and cardiology teams and the patient, taking into account disease stage, competing cancer and cardiovascular risks, and alternative treatment options. We recommend that for patients with non-severe cardiotoxicity, BRAF inhibitor therapy may continue unchanged but MEK inhibitor therapy should be at least temporarily withheld.^63^, ^64^, ^65^, ^66^, ^67^, ^68^ Repeat echocardiography should be carried out after 4 weeks and, if LVEF has improved to greater than the lower limit of normal, the MEK inhibitor may be restarted at a reduced dose. However, if the LVEF does not improve, there should be consideration for permanent discontinuation of the MEK inhibitor with multidisciplinary discussion. In cases of severe toxicity, the MEK inhibitor should be permanently discontinued.^10^ It is suggested that, in the context of BRAF inhibitor/MEK inhibitor–associated LVSD, treatment with BRAF inhibitor monotherapy may be continued. However, we also suggest that in patients with symptomatic heart failure, consideration should be made to stopping both agents at least temporarily and only restarted when LVEF has recovered and symptoms have resolve. It should be noted that long-term BRAF inhibitor monotherapy may be undesirable for other reasons, including treatment resistance and skin toxicities, and that certain BRAF inhibitors such as encorafenib are only licensed for use in combination with MEK inhibitors.

Evidence of the reversibility of LVSD following cessation of MEK inhibitor and commencement of heart failure therapies is currently limited but, similar to cardiotoxic effects of other tyrosine kinase inhibitor anti-cancer therapies,^71^^,^^72^ there appears to be a reversible component. A retrospective, observational study by Berger et al^73^ examined 88 patients treated with BRAF inhibitor and MEK inhibitor. Twelve (17.4%) patients on combination therapy experienced a reduction in LVEF (defined as reduction in LVEF ≥10% from baseline to a value <55%). Treatment with angiotensin-converting enzyme (ACE) inhibitors and beta-blockers was associated with a greater recovery in LVEF than in those not commenced on cardioprotective medications. It is not possible to draw conclusions from this small observational report, and there are no guidelines or international consensus documents regarding the management of BRAF inhibitor/MEK inhibitor–associated LVSD. However, we recommend the introduction of an ACE inhibitor, or angiotensin receptor blocker in patients who are intolerant of ACE inhibitors because of cough, and beta-blocker (carvedilol or bisoprolol) in patients with asymptomatic BRAF inhibitor/MEK inhibitor–associated LVSD. This is in line with recommendations on the management of LVSD during cancer therapy outlined in the 2016 European Society of Cardiology position paper.^69^ Patients with symptomatic heart failure should be treated with usual evidence-based heart failure therapies.^57^ Patients with heart failure or LVSD should be jointly managed by both oncology and cardiology teams, ideally with involvement of a specialist cardio-oncology service. There are no guidelines on long term management of BRAF inhibitor/MEK inhibitor–associated LVSD. We recommend that patients with symptomatic heart failure or asymptomatic patients with any degree of ongoing LV systolic impairment should continue treatment with heart failure therapies on a long-term basis. Decisions regarding continuation of ACE inhibitor and beta-blocker in asymptomatic patients with recovery of LVEF on cessation of BRAF inhibitor and MEK inhibitor are challenging, and there should be an individualized approach taking into account the patient’s wishes. However, our own practice is to continue ACE inhibitor and beta-blocker therapy in the long term in these patients.

While LVSD is reported in clinical trials,^14^^,^^16^^,^^22^^,^^55^^,^^56^ there is no description of the timing of onset or natural history of LV systolic dysfunction following treatment with BRAF inhibitor and MEK inhibitor. Prospective studies are required to fully characterize the time course, appropriate monitoring, and reversibility of BRAF inhibitor– and MEK inhibitor–induced LVSD. Additionally, the utility of cardiac biomarkers (especially natriuretic peptides and troponin) for baseline risk and longer-term monitoring for cardiotoxic effects of these drugs has not been validated prospectively.

#### Pathophysiologic mechanisms of BRAF and MEK inhibitor–associated LV dysfunction

The underlying mechanisms by which LVSD occurs in association with BRAF inhibitor and MEK inhibitor has not been extensively studied. It is to be expected that disruption of the MAPK pathway could lead to a change in the physiological cardioprotective mechanisms described previously and affect apoptosis, remodeling, and hypertrophy, ultimately leading to LVSD.^36^^,^^74^ In mouse models, ERK null mice have normal cardiac size and function but are rendered more susceptible to a subsequent cardiac insult.^45^ This supports the hypothesis whereby a “second hit” such as hypertension or ischemia is required to provoke a reduction in LVEF after exposure to BRAF inhibitor/MEK inhibitor. Additionally, inhibition of MEK1/2 in smooth muscle cells leads to reduced cell proliferation and other processes essential for angiogenesis.^75^ Consequences of this may include cardiac microvascular rarefaction and a consequent impairment of adaptation to increased cardiac afterload. These putative myocardial effects are particularly pertinent to the cardiovascular adverse effect profile of BRAF/MEK inhibition. Indeed, BRAF/MEK inhibitor–induced hypertension and the consequent elevation in cardiac afterload may amplify their direct cardiotoxic effects.

### Key Points

• The incidence of BRAF inhibitor/MEK inhibitor–associated LVSD reported in clinical trials is 2% to 12%, but this may be an underestimate.
• Reduction in LVEF is more common in patients treated with combination BRAF inhibitor/MEK inhibitor therapy than BRAF inhibitor monotherapy.
• Echocardiography surveillance is recommended at baseline, after 4 weeks, and at 3-monthly intervals thereafter throughout treatment.
• In the event of BRAF inhibitor/MEK inhibitor–associated LVSD consideration should be made to at least temporarily withholding MEK inhibitor, with or without alteration of BRAF inhibitor therapy, and commencing potentially cardioprotective therapies including ACE inhibitor and beta-blocker. Decisions should be made jointly by oncology and cardiology teams taking into account disease stage and alternative treatment options.

### Hypertension

#### Incidence of BRAF inhibitor/MEK inhibitor–associated hypertension

Hypertension is the most common adverse event reported with BRAF inhibitor and MEK inhibitor, and the incidence is higher with combination therapy.^16^ A meta-analysis including 5 randomized clinical trials reported an increased risk of systemic hypertension (RR: 1.49; 95% CI: 1.12-1.98]) that occurred in 19.5% of patients treated with combination of BRAF inhibitor and MEK inhibitor compared with 14% in the BRAF inhibitor monotherapy group.^9^ There are no previously described risk factors associated with an increased risk of BRAF inhibitor– and MEK inhibitor–associated hypertension. Clinical trial reporting of hypertension is based on CTCAE definitions of severity (grade 1: systolic blood pressure [SBP] 120-139 mm Hg or diastolic blood pressure [DBP] 80-89 mm Hg; grade 2: SBP 140-159 mm Hg or DBP 90-99 mm Hg; grade 3: SBP ≥160 mm Hg or DBP ≥100 mm Hg; grade 4: elevated blood pressure with life-threatening consequences).

In the COMBI-d trial, “all-grade” hypertension was reported in 22% of patients receiving dabrafenib and trametinib in combination (4% grade 3) versus 14% of patients receiving dabrafenib alone.^16^ In the coBRIM trial, all-grade hypertension was reported in 15.8% of patients receiving vemurafenib and cobimetinib combination (6% grade ≥3) versus 8% of those receiving vemurafenib alone.^55^ In the COLUMBUS trial, the incidence of all-grade hypertension was 11% in those receiving encorafenib and binimetinib, with grade ≥3 hypertension reported in 6% of patients.^56^

#### Assessment and management of blood pressure before and during BRAF inhibitor/MEK inhibitor treatment

In the absence of guidelines, the monitoring and management of BRAF inhibitor and MEK inhibitor–associated cardiovascular toxicity remains based on expert opinion. In a practical overview of multisystem toxic effects of BRAF inhibitor/MEK inhibitor therapy, Welsh and Corrie^10^ provide suggestions for the management of BRAF inhibitor– and MEK inhibitor–associated hypertension. They recommend that blood pressure is monitored every cycle with target SBP <140 mm Hg systolic and DBP <90 mm Hg. During BRAF inhibitor/MEK inhibitor treatment, if this is not achievable despite antihypertensive medications, the dose of MEK inhibitor should be reduced. For patients with SBP ≥160 mm Hg or DBP ≥100 mm Hg, it is recommended that MEK inhibitor is stopped and restarted at a reduced dose when BP is <140/90 mm Hg. We recommend that in cases of recurrent hypertension, the MEK inhibitor dose may be reduced a second time but should be discontinued in the event of further hypertension recurrence. In the case of grade 4 hypertension (ie, hypertension with life-threatening consequences), the MEK inhibitor should be permanently discontinued, BRAF inhibitor should be temporarily stopped and hypertension treated on an urgent basis.^10^ Again, decisions regarding the temporary or permanent cessation of the MEK inhibitor should be made following multidisciplinary discussion between oncology and cardiology or cardio-oncology teams and the patient, and taking into account disease stage and patient prognosis.

**Figure 3:**
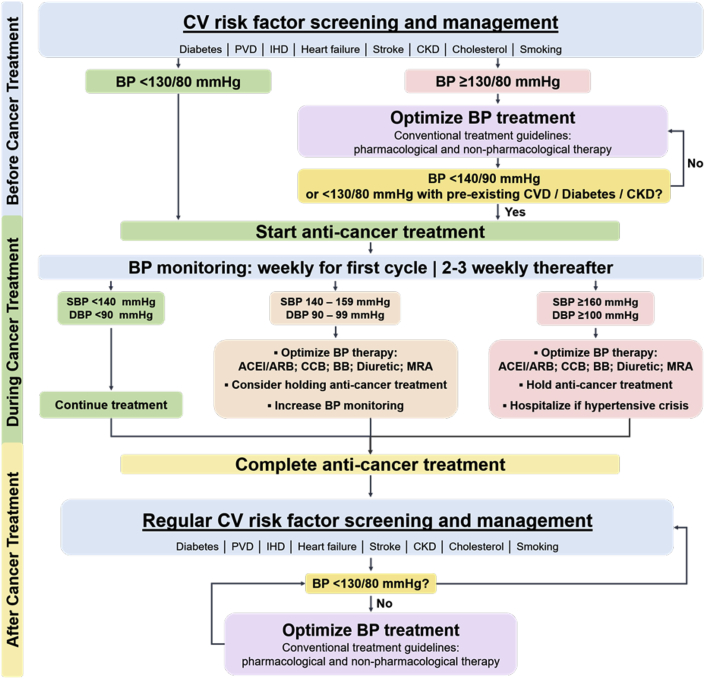
Screening, Monitoring, and Treatment of BP During Anti-Cancer Therapy We propose blood pressure (BP) targets for patients before, during, and after treatment with systemic anti-cancer therapies associated with hypertensive effects. Cardiovascular risk factor screening should be performed before and on completion of anti-cancer treatment. An ideal BP target of <130 mm Hg prior should be achieved prior to anticancer therapy. During cancer therapy, BP should be monitored frequently and antihypertensive therapy commenced when BP exceeds 140/90 mm Hg. Patients with BP >140/90 mm Hg should have BP optimized with angiotensin-converting enzyme inhibitor (ACEi) or angiotensin II receptor blocker (ARB), calcium-channel blocker (CCB), beta-blocker (BB), diuretic or mineralocorticoid receptor antagonist (MRA), and enhanced BP monitoring. On completion of anti-cancer treatment, a long-term BP target of <130/80 mm Hg is recommended. Reprinted with permission from van Dorst et al.^76^ CKD = chronic kidney disease; CV = cardiovascular; CVD = cardiovascular disease; DBP = diastolic blood pressure; IHD = ischemic heart disease; PVD = peripheral vascular disease; SBP = systolic blood pressure; other abbreviations as in

Given that BRAF inhibitors and MEK inhibitors are increasingly used in the adjuvant setting, adequate monitoring and treatment of hypertension is of even greater importance to reduce the risk of developing cardiovascular disease in the longer term. We have recently published strategies to screen, monitor, and treat hypertension in the oncology population. We have made suggestions for BP targets for patients before, during, and after treatment with systemic anti-cancer therapies associated with prohypertensive effects.^76^ These include an ideal blood pressure target of <130/80 mm Hg before anticancer therapy, and this is particularly relevant for patients treated in the adjuvant setting. During cancer therapy, in agreement with recommendations from Welsh and Corrie,^10^ we suggest that antihypertensive therapy should be commenced when BP exceeds 140/90 mm Hg (Figure 3).

We recommend that regular screening for cardiovascular risk factors should continue following the completion of anti-cancer therapy. When BRAF inhibitors and MEK inhibitors are used in the setting of advanced disease, BP targets may be more lenient and balanced against the limited survival prognosis for this patient group. However, BP control remains important to avoid end-organ effects that might compromise the ability to safely deliver optimal cancer therapy. When treatment with BRAF inhibitor/MEK inhibitor is in the adjuvant setting, we would recommend strict monitoring and management to target BP levels as outlined in Figure 3.^76^

There is no specific evidence to suggest one class of antihypertensive agent over another in the management of BRAF inhibitor/MEK inhibitor–associated hypertension, but we suggest that ACE inhibitors or angiotensin receptor blockers should be used in patients with coexisting LVSD or proteinuria and in the absence of contraindications. Nondihydropyridine calcium-channel blockers (verapamil and diltiazem) should be avoided because of their potential to increase plasma concentrations of certain MEK inhibitors and BRAF inhibitors.

#### Mechanisms of BRAF and MEK inhibitor–associated hypertension

Reduced bioavailability of NO may be implicated in the development of BRAF/MEK inhibitor–associated hypertension. BRAF and MEK inhibition is associated with upregulation of expression of CD47 in melanoma cells in vitro. CD47 inhibits NO/cyclic guanosine monophosphate signaling leading to a reduction in NO bioavailability with consequent vasoconstriction and hypertension. However, this potential pathophysiologic mechanism has not been tested in endothelial or vascular smooth muscle cells,^77^ nor in vivo. A small preclinical study in mice suggested that MEK/ERK signaling may be required for central or sympathetic regulation of blood pressure.^78^

### Key Points

• Hypertension is the most commonly reported CVAE associated with BRAF and MEK inhibitors, and the incidence is higher with combination therapy versus BRAF inhibitor monotherapy.
• BP should be monitored with every cycle of treatment.
• During BRAF inhibitor/MEK inhibitor treatment antihypertensive therapy should be commenced when BP exceeds 140/90 mm Hg or 130/80 mm Hg in patients with pre-existing CV risk factors or disease, with an ideal target of <130/80 mm Hg before anticancer therapy.
• BP targets should take into account disease stage and prognosis with strict monitoring and treatment to the BP target advised in the adjuvant setting.

### Arrhythmia

Treatment with BRAF inhibitor and MEK inhibitor is associated with a risk of atrial arrhythmias and electrocardiographic QT interval prolongation. Systematic capture and reporting of arrhythmias are not common in clinical trials of BRAF inhibitor/MEK inhibitor; therefore, good data on this adverse effect are lacking. In the small number of studies that did report arrhythmia, the most common was AF. In the analysis of the Truven Health Analytics/IBM MarketScan database, AF was reported in 2.7% of patients^59^ and was also reported in 1.5% of patients treated with vemurafenib in an open-label safety study.^79^^,^^80^ In a meta-analysis, the RR of AF was not increased by combination therapy when compared with BRAF inhibitor alone.^9^ Patients who develop AF while being treated with BRAF inhibitor/MEK inhibitor should be managed in accordance with standard cardiology guidelines, although caution is required when considering anticoagulation, as concurrent treatment with MEK inhibitors^66^, ^67^, ^68^ and certain BRAF inhibitors^64^ may be associated with an increased risk of bleeding. There are no guidelines for the monitoring of arrhythmia while on treatment with BRAF inhibitor and MEK inhibitor. We suggest that monitoring should be guided by patient-reported symptoms and would include ECG and multiday ECG in the first instance.

QTc interval prolongation, defined as an increase of more than 60 ms compared with baseline or a QTc interval >500 ms,^10^ has been associated with the BRAF inhibitors vemurafenib and dabrafenib. It is not generally associated with MEK inhibitors.^14^^,^^79^^,^^81^ In a multicenter safety study of vemurafenib, QTc interval prolongation was observed in 2% of patients with a median time to development of 1.9 months.^79^ A similar incidence (3%) was observed in patients treated with dabrafenib in a phase II trial.^81^ In the COLUMBUS trial, the incidence of QTc interval prolongation was 0.5% in patients treated with a encorafenib and was not increased by combination with binimetinib.^56^ It is not recommended to commence BRAF inhibitors in patients with baseline QTc interval >500 ms or those with uncorrected electrolyte abnormalities or patients who cannot safely discontinue concurrent medications known to prolong the QT interval. It is advised to record a baseline electrocardiogram and to check serum electrolytes in all patients to be commenced on BRAF inhibitor. These should be repeated after 1 month of treatment and after any dose adjustments. During treatment, if QTc interval measures >500 ms or there is an increase of >60 ms from baseline, then BRAF inhibitor should be permanently discontinued. In the case of a QTc interval increase of >60 ms but with an absolute QTc duration <500 ms, treatment should be interrupted and restarted at a lower dose once the QTc interval returns to within 60 ms from baseline. Other risk factors for QT interval prolongation should be addressed concurrently.^10^ It is important to note that although there is a clear incidence of QTc interval prolongation associated with BRAF inhibition, there is no strong evidence to suggest that this is closely correlated with an increased risk of torsade de pointes, which is described rarely.

The pathophysiologic mechanisms of BRAF inhibitor– and MEK inhibitor–associated QTc prolongation and atrial arrhythmia are not known.

### Key Points

• BRAF inhibitors are associated with a 1% to 5% incidence of QTc interval prolongation.
• AF is the most commonly reported arrhythmia associated with BRAF inhibitor/MEK inhibitor with an incidence of 1% to 4%.
• ECG is recommended at baseline, after 1 month of treatment, and after any dosage change.

### Thromboembolism

As well as contributing to recurrent vasoconstriction and hypertension, BRAF inhibitor/MEK inhibitor–related reductions in NO bioavailability also lead to an imbalance between thrombotic and antithrombotic states and may result in thrombotic sequelae. Meta-analysis of 5 randomized controlled trials including 2,317 patients with melanoma concluded that combination therapy with BRAF inhibitor and MEK inhibitor was associated with a higher risk of pulmonary embolism (RR: 4.36; 95% CI: 1.23-15.45; *P =* 0.02) compared with BRAF inhibitor monotherapy. The incidence of pulmonary embolism was 2.2% in the combination group compared with 0.4% in the BRAF inhibitor monotherapy group.^9^ A “real-world” observational, nonrandomized analysis of the Food and Drug Administration’s adverse reporting system^59^ including 7,712 adverse events also reported an increased risk of venous thromboembolism (OR: 1.8; 95% CI: 1.12-2.89) in combination therapy compared with BRAF inhibitor monotherapy. Pulmonary embolism accounted for 1% of reported events and venous thromboembolism for 1.1% of events and was more common with combination therapy.

Reporting of arterial thrombosis in clinical trials is limited. The incidence of myocardial infarction in the coBRIM trial was 0.4%^55^ in the BRAF inhibitor/MEK inhibitor group versus 0% in the BRAF inhibitor plus placebo group. A similar incidence of 1% was reported in the COLUMBUS trial for patients treated with BRAF inhibitor plus MEK inhibitor.^56^ Meta-analysis comparing combination therapy versus BRAF inhibitor monotherapy concluded that the risk ratio of myocardial infarction was similar in both groups.^9^

There are no guidelines regarding screening or monitoring for thromboembolism in patients treated with BRAF inhibitor and MEK inhibitor. If thromboembolism is suspected, then local diagnostic pathways should be followed. The pathophysiologic mechanisms resulting in BRAF inhibitor– and MEK inhibitor–associated thromboembolism are not known.

### Key Points

• BRAF inhibitor and MEK inhibitor combination therapy is associated with an increased risk of venous thromboembolism when compared with BRAF inhibitor monotherapy.
• Pulmonary embolism has been reported in 1% to 2% of patients treated with BRAF inhibitor/MEK inhibitor

### BRAF inhibitors and MEK inhibitors in combination with immunotherapy

Although BRAF inhibitors and MEK inhibitors are effective in improving overall survival in patients with BRAF-mutated melanoma, a concern remains over the development of resistance and subsequent disease progression. An alternative treatment option is the use of immunotherapy agents such as ipilimumab (CTLA-4 blockade), nivolumab and pembrolizumab (PD-1 blockade).^82^^,^^83^ Although immunotherapy agents can lead to a more durable response, the overall response rate is lower when compared with BRAF inhibitors and MEK inhibitors.^17^ The different strengths of these approaches has provoked interest in the potential benefits of combining targeted therapy and immunotherapy in patients with advanced metastatic or unresectable melanoma.

The tumor environment in BRAF-mutated melanoma exhibits multiple acquired immunosuppressive features. These include low T cell infiltrates, increased regulatory T cells, increased myeloid-derived suppressor cells, and immature antigen presenting cells.^84^ Preclinical studies using mouse models and melanoma cells lines have shown that these unfavorable immunologic features are reversed by BRAF inhibitor and MEK inhibitor.^85^ Biopsies from patients treated with BRAF inhibitor and MEK inhibitor who have disease progression show a decrease in T cell infiltration and activity and an increase in inhibitory molecule expression, which may represent an immune-mediated resistance to therapy.^86^ The combination of targeted therapy and immunotherapy to treat BRAF-mutated melanoma to improve antitumor immunity and reduce immune-mediated resistance is therefore an attractive potential therapeutic option.

Preclinical studies in mouse models have shown a beneficial effect on the tumor environment with combination therapy with improved tumor responses.^87^ A phase 2 randomized trial (KEYNOTE-022 [A Study of the Safety and Efficacy of Pembrolizumab (MK-3475) in Combination With Trametinib and Dabrafenib in Participants With Advanced Melanoma]) enrolled 120 patients with BRAF-mutated melanoma (largely stage IV) to compare the efficacy of the combination of pembrolizumab, dabrafenib and trametinib versus dabrafenib, trametinib plus placebo.^88^ Despite demonstrating a durable response and encouraging progression-free survival with the addition of immunotherapy to BRAF inhibitor/MEK inhibitor, this failed to meet statistical significance. This trial did highlight an increased risk of treatment related toxicities with triple combination therapy. Grade 3 to 5 adverse events occurred in 70% of the triple therapy group compared with 45% of the placebo group. Of note, the incidence of grade 3 hypertension was 8.3% in the triple therapy group compared with 3.3% in the placebo group. This poses a concern over the potential increased risk of cardiovascular toxicity with combination therapies, particularly the potential for a “double-hit” phenomenon of hypertension in the potentiation of LVSD and heart failure. A subsequent phase 3 randomized trial IMspire 150^19^ assessed the use of anti-PD-L1 monoclonal antibody atezolizumab in combination with vemurafenib and cobimetinib versus placebo and has shown significantly prolonged progression-free survival in the treatment group. In contrast to the KEYNOTE-022 trial, the addition of atezolizumab did not increase the prevalence of treatment related grade 3 to 4 adverse events (79% vs 73% in the control group) and did not escalate typical BRAF inhibitor/MEK inhibitor–associated adverse events.

Several randomized phase 3 trials examining a variety of combinations of immunotherapy plus targeted therapy are ongoing, including ImmunoCobiVem (Evaluating the Efficacy and Safety of a Sequencing Schedule of Cobimetinib Plus Vemurafenib Followed by Immunotherapy With an Anti- PD-L1 Antibody in Patients With Unresectable or Metastatic BRAF V600 Mutant Melanoma; NCT02902029), SECOMBIT (Sequential Combo Immuno and Target Therapy Study; NCT02631447), and DREAMseq (Dabrafenib and Trametinib Followed by Ipilimumab and Nivolumab or Ipilimumab and Nivolumab Followed by Dabrafenib and Trametinib in Treating Patients With Stage III-IV BRAFV600 Melanoma; NCT02224781) trials, and part 3 of COMBI-I trial (A Study of the Anti-PD-1 Antibody PDR001, in Combination With Dabrafenib and Trametinib in Advanced Melanoma). These trials not only inform optimal combinations and sequences of treatment, but also will contribute to the characterization of potential adverse effects of combination treatment. This is increasingly relevant, as prolonged survival results in patients continuing treatment with BRAF inhibitor and MEK inhibitor for longer periods and the long-term CVAEs are poorly characterized.

### Key Points

• The combination of BRAF inhibitor/MEK inhibitor with immunotherapy may be a therapeutic option to reduce immune-mediated resistance in BRAF-mutated melanoma.
• Ongoing phase 3 trials are essential to inform treatment strategy and characterize the adverse effects of combination treatment.

## Conclusions

BRAF and MEK inhibitors have revolutionized treatment for patients with melanoma. However, they are associated with CVAEs including systemic hypertension, LVSD, arrhythmias, and venous thromboembolism. The mechanisms underlying these cardiovascular toxicities are incompletely understood, and the reporting of adverse cardiovascular events in clinical trials is variable. Systemic hypertension and LVSD are the most commonly reported CVAEs in clinical trials, and their incidence is higher with the use of combination BRAF inhibitor/MEK inhibitor therapy compared with BRAF inhibitor monotherapy. Evidence to guide the best surveillance and treatment of BRAF inhibitor– and MEK inhibitor–associated cardiovascular effects is currently limited.

Better understanding of the mechanisms and incidence of BRAF and MEK inhibitor–associated cardiovascular toxicity is vital. This will inform better risk stratification and cardiovascular surveillance for patients receiving these medications with the aim of limiting temporary or permanent cessation of treatment and maximizing their anti-cancer potential. Increased reporting of CVAEs in clinical trials and further research into the mechanisms underlying these cardiotoxic effects ought to provide important information to allow optimal anti-cancer utility of BRAF and MEK inhibitors while minimizing potential cardiovascular adverse effects.

## Funding Support and Author Disclosures

Drs Glen and Lang are supported by an unrestricted grant from Roche Diagnostics, Switzerland. Drs Petrie and Lang are supported by a British Heart Foundation Centre of Research Excellence Grant (RE/18/6/34217). Dr Evans has received advisory board or speaker fees from AstraZeneca, Bayer, Britsol-Myers Squibb, Clovis, Medivir, Merck Sharp & Dohme, Nucana, and Roche/Genentech; and has received research grant funding from Adaptimmune, Astellas, Bayer, Boehringer Ingelheim, Celgene, Codiak, Esai, GlaxoSmithKline, iOncture, Johnson and Johnson, Merck Sharp & Dohme, Novartis Pfizer, and Roche/Genentech. Dr Jones has received personal fees from Astellas, AstraZeneca, Bristol-Myers Squibb, Bayer, Exelixis, Janssen, Ipsen, Merck Serono, Merck Sharp & Dohme, Novartis, Pfizer, Roche, Sanofi Genzyme, and EUSA; and research grant funding from Astellas, AstraZeneca, Bayer, Exelixis and Roche. Dr Petrie has received research funding from Boehringer Ingelheim, Roche, SQ Innovations, AstraZeneca, Novartis, Novo Nordisk, Medtronic, Boston Scientific, Pharmacosmos, 3R LifeSciences; and served as a consultant and on clinical trials committees for Boehringer Ingelheim, Novartis, Roche, Corvia, AstraZeneca, Novo Nordisk, Medtronic, AbbVie, Bayer, Takeda, Cardiorentis, Pharmacosmos, and Siemens. Dr Lang has received speaker fees from Roche, Pfizer, and Novartis. All other authors have reported that they have no relationships relevant to the contents of this paper to disclose.
